# Deltopectoral Flap in the Era of Microsurgery

**DOI:** 10.1155/2014/420892

**Published:** 2014-01-02

**Authors:** R. C. L. Chan, J. Y. W. Chan

**Affiliations:** Division of Head and Neck Surgery, Department of Surgery, Queen Mary Hospital, 102 Pokfulam Road, Hong Kong

## Abstract

*Background*. Our study aimed to review the role of deltopectoral (DP) flap as a reconstructive option for defects in the head and neck region in the microvascular era. *Methods*. All patients who received DP flap reconstruction surgery at the Department of Surgery, Queen Mary Hospital, between 1999 and 2011 were recruited. Demographic data, indications for surgery, defect for reconstruction, and surgical outcomes were analyzed. *Results*. Fifty-four patients were included. All but two patients were operated for reconstruction after tumour resection. The remaining two patients were operated for necrotizing fasciitis and osteoradionecrosis. The majority of DP flaps were used to cover neck skin defect (63.0%). Other reconstructed defects included posterior pharyngeal wall (22.2%), facial skin defect (11.1%), and tracheal wall (3.7%). All donor sites were covered with partial thickness skin graft. Two patients developed partial flap necrosis at the tip and were managed conservatively. The overall flap survival rate was 96.3%. *Conclusions*. Albeit the technical advancements in microvascular surgery, DP still possesses multiple advantages (technical simplicity, reliable axial blood supply, large size, thinness, and pliability) which allows it to remain as a useful, reliable, and versatile surgical option for head and neck reconstruction.

## 1. Background

The deltopectoral (DP) flap, also called by some as the Bakamjian flap [1], was actually first described by Aymard in 1917 [2]. Aymard described raising a medially based fasciocutaneous flap from the shoulder skin which was then tubed and used for staged nasal reconstruction [2]. In 1931, Joseph, using illustrations of Manchot from 1889, justified and published illustrations of DP flaps as vascularized pattern flaps [3, 4].

The DP flap, however, did not spark much interest until it was reintroduced by Bakamjian in 1965. Bakamjian reported the use of DP for pharyngoesophageal reconstruction after laryngopharyngectomy [1]. The DP flap became the “workhorse” flap for head and neck reconstruction and enjoyed great popularity in the 1960s, but its popularity gradually faded out with the advent of pedicled myocutaneous flaps and microvascular free flaps.

DP flap is thin and pliable and has excellent colour and texture match with the head and neck area. Its reliable anatomy allows quick and easy harvest. A large flap can be harvested, especially with a delayed procedure. Its donor site has minimal functional deficit and can be easily concealed. The flap can be used even in patients who had previous pectoralis major flap if skin of the DP flap was not cut into during the harvest of the pectoralis flap. Its pedicle can be divided and returned to minimize donor site morbidity and improve cosmetic outcomes of both donor and recipient sites.

Skin grafting is usually necessary for donor site coverage, with the exception of small defect in patients with lax skin. In female patients, the scarring may also lead to breast asymmetry and nipple distortion. Distal flap necrosis is not uncommon if the skin paddle was extended too much into the deltoid region without a delay procedure. Hirsute skin may be troublesome for patients but hair removal (e.g., with laser) can always be performed later.

The aim of this study is to review the role of deltopectoral (DP) flap as a reconstructive option for defects in the head and neck region in the microvascular era.

## 2. Methods

All patients who received DP flap reconstruction surgery at the Department of Surgery, Queen Mary Hospital between January 1999 and December 2011 were recruited. A retrospective chart review was performed to collect information on patient demographics, indications for surgery, defects for reconstruction, surgical techniques, donor site complications, and recipient site complications.

All flaps were harvested as fasciocutaneous flaps based on second and third perforators of the internal mammary artery arising from the deltopectoral groove. The skin medial to the DP groove is reliably nourished by the internal perforators, but the skin lateral to the DP groove is usually nourished by musculocutaneous perforators arising from the deltoid muscles. The extended portion of DP flap beyond the DP groove is therefore essentially a random pattern flap. Whenever this was necessary, we limited our extension within the 1 : 1 base-to-length ratio.

The flaps were designed by estimating the arc of rotation needed to reach the defect and harvested in a lateral-to-medial fashion. We routinely incorporate the deltopectoral fascia into the flap and perform the dissection in a subfascial plane. Care was taken to keep at least 2 cm from the lateral border of sternum to avoid injury of the pedicle.

The inset depended on the defect. For neck or lower face defects, the skin between the defect and donor site may be excised allowing one-stage reconstruction. Alternatively, the skin bridge could be left intact and have the DP flap tubed over the neck skin instead. The latter would require a second stage division of pedicle with or without returning the pedicled component. This could be performed under local anaesthesia.

For staged reconstruction for circumferential pharyngeal defects, we used DP flap for posterior wall reconstruction in the first stage. The anterior walls were typically reconstructed with pectoralis major flap after a 2- to 4-week delay. Skin incisions were made on the DP flap along the edges of the neopharynx to be reconstructed. The edges of the DP flaps were then sutured to the skin island of the pectoralis major flap to minimize the chance of leak.

For terminal tracheostomy with short tracheal remnant, DP flaps were inset to augment the posterior tracheal wall in a one-stage reconstruction.

The donor sites were all covered with partial thickness skin graft.

## 3. Results

Fifty-four patients were included over the 13-year study period. The median age was 60 years with a range from 37 to 99 years. There were 42 males and 12 females. The median follow time was 30 months. All but two patients were operated for reconstruction after tumour resection. The remaining two patients were operated for necrotizing fasciitis and osteoradionecrosis. None of the flaps was delayed. The mean length and width were 16.3 cm (standard deviation (SD) 2.0 cm) and 8.4 cm (SD 1.8 cm), respectively.

The majority of DP flaps were used to cover neck skin defect (63.0%). Other reconstructed defects included posterior pharyngeal wall (22.2%), facial skin defect (11.1%), and tracheal wall (3.7%). All donor sites were covered with partial thickness skin graft.

Two patients developed partial flap necrosis at the tip and were managed conservatively with regular dressing. There was no complete flap failure. The overall flap survival rate was 96.3%. All donor site wounds healed uneventfully.

## 4. Discussion

Very few flaps possess the advantages that DP flap can offer: a thin, pliable pedicled flap with minimal donor site morbidity and excellent colour/texture match to head and neck area. Bulkiness of pedicled myocutaneous flap can be cosmetically unpleasant and can limit its rotation and hence the versatility in reconstruction. Free radial forearm flap offers a thin, pliable flap but at the cost of a significant functional and cosmetic disturbance. It also requires microvascular anastomosis.

Over the last few decades, several variations of flap harvesting have been developed which further enhance its versatility. Pedicled island flap is a modification of the conventional harvesting technique that allows single stage insetting by creating an island flap. Lash first described this method by deepithelializing the skin bridge and tunneling the fascial pedicle subcutaneously [5]. Subsequently, it was further modified by placing a single incision from second intercostal space to the midpoint of the skin paddle. Superior and inferior skin flaps were then developed in the subdermal plane preserving the vascular pedicle underneath. The flap dissection then proceeded from lateral to medial direction in the subfascial plane [6, 7]. Such techniques allow a greater axis of rotation, more versatility of flap inset, and better cosmetic outcome in both donor and recipient sites and avoided or at least minimized the need to skin graft the donor site. In the review of 16 island DP flap procedures, Mortensen and Genden reported a 100% flap survival rate indicating that such modification is safe and reliable [6].

Several techniques were described for utilizing DP flap to cover a defect with two epithelial surfaces. Bakamjian himself described a two-staged L-shaped DP flap: a short limb of the “L” extending down the proximal upper arm was raised and folded under the deltoid skin as a buried skin flap in the initial stage. After the delay, the DP flap, now with two epithelial surfaces, was then raised and transferred to the defect [8]. Alternatively, the DP flap can be split longitudinally at its distal end to provide the coverage of a defect with two epithelial surfaces, as described by Krizek and Robson [9]. This allows one-stage reconstruction of a defect with two epithelial surfaces. It is a safer design compared to folding the flap over itself in terms of flap vascularity. Another way to reconstruct a double epithelial surface defect with DP flap is to skin graft the undersurface.

Improvement of microsurgical techniques over the last few decades allowed us to harvest DP flap as an internal mammary artery perforator (IMAP) flap [10, 11]. Perforators were located with hand-held Doppler preoperatively. The flap is fashioned in the usual manner until the perforators are identified. They are then dissected through the intercostal muscles. Costal cartilages are often removed to enhance exposure. This technique can be applied to the harvest of both pedicled and free IMAP flap.

**Figure 1:**
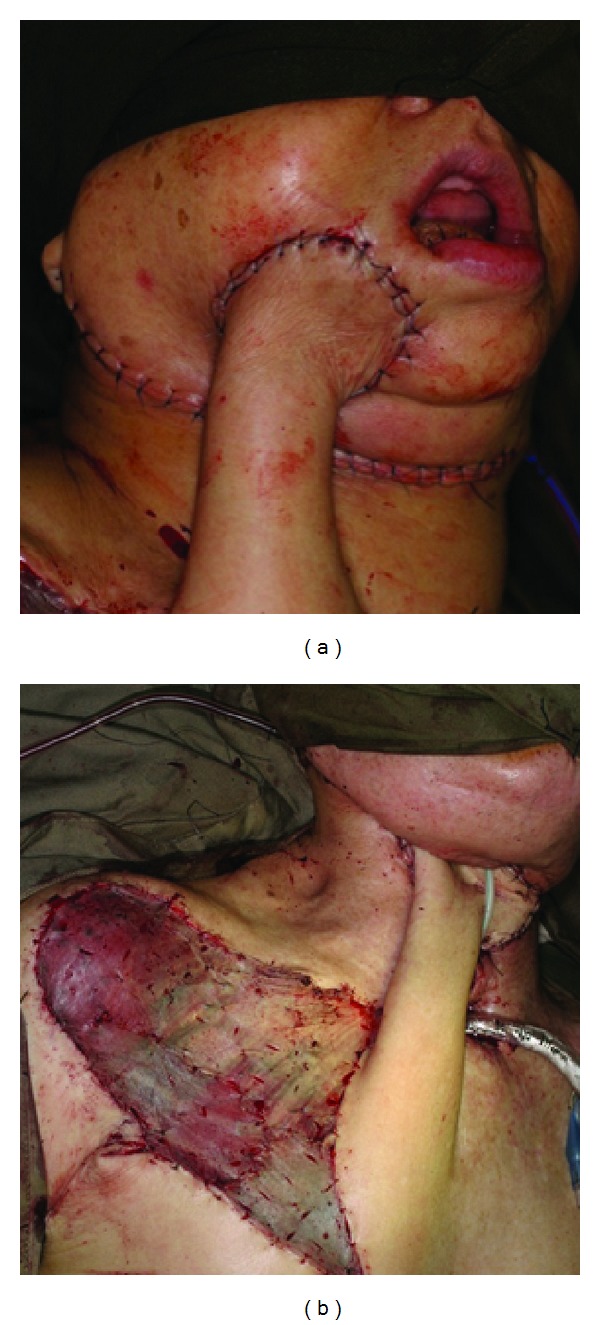
(a) Tubed DP flap for reconstruction of facial skin defect. (b) Tubed DP flap for reconstruction of neck skin defect. DP can be divided two weeks after initial surgery under local anaesthesia for both cases.

**Figure 2:**
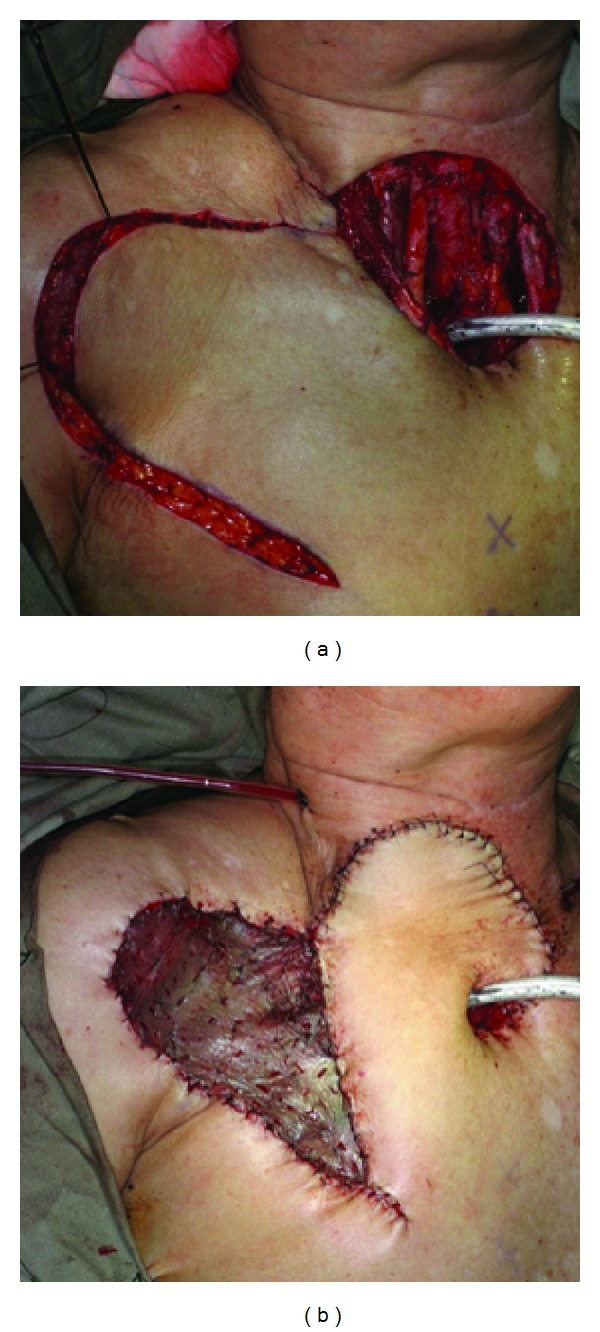
(a) Recurrent laryngeal carcinoma treated with total laryngectomy and skin resection. (b) DP flap covered the neck skin defect and augmented the posterior tracheal wall.

**Figure 3:**
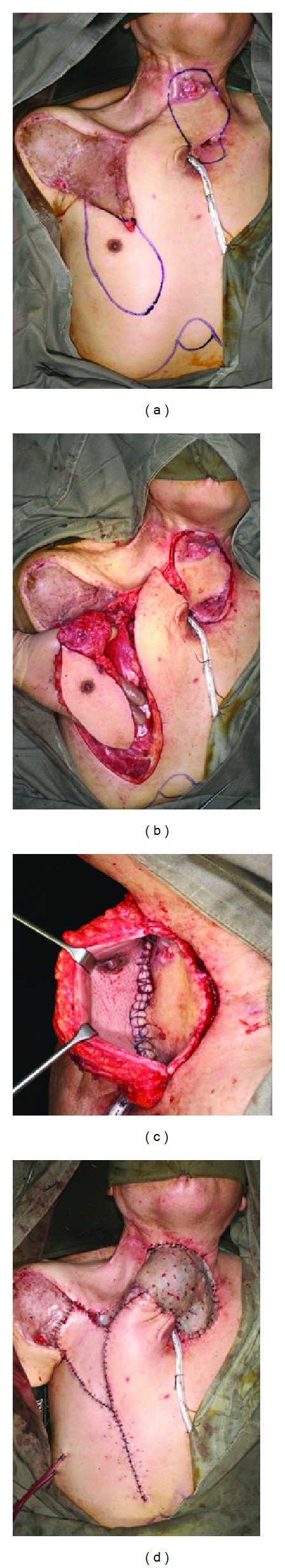
(a) Total laryngectomy and circumferential pharyngectomy defect in a patient unfit for free flap reconstruction. Posterior pharyngeal wall previously reconstructed with DP flap. (b) DP flap divided. Edges of neopharyngeal wall mobilized. Pectoralis major (PM) flap raised for anterior pharyngeal wall reconstruction. (c) Neopharynx reconstructed with divided DP flap as posterior wall and PM flap as anterior wall. (d) Undersurface of PM flap covered with skin graft. Base of previous DP flap used to protect anastomosis and prevent salivary spillage into the tracheostomy in case of leakage. Axillary flap was also raised to cover the chest wall defect in this patient.

**Figure 4:**
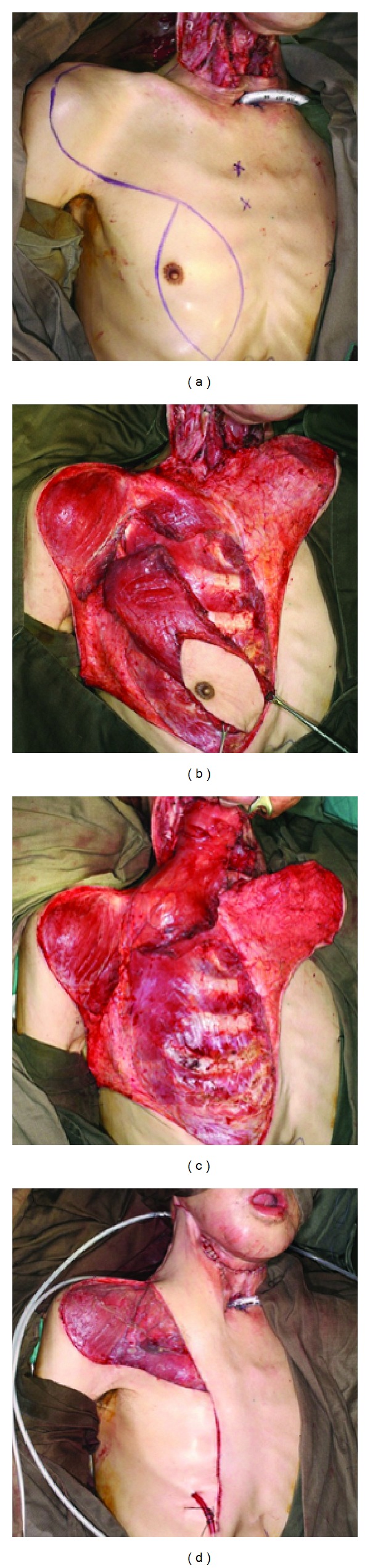
Simultaneous use of DP and pectoralis major (PM) flaps in a patient with partial laryngectomy and neck skin defects. (a) Incisions of skin island of PM flap planned so as to preserve the intercostal perforators supplying the DP flap. (b) Both flaps are raised. (c) PM flap covered the partial pharyngectomy defect; the muscle bulk also protected the major vessels in the neck in case of any leakage. (d) DP flap covered the neck skin defect. Donor site covered with skin graft.

Despite the advent of microsurgery, we have found DP flap to be very useful in various clinical scenarios. The flap is most appropriate in patients who required reconstruction of the lower face or neck region but are not ideal candidates for free flap reconstruction (Figure 1). It also provided a quick and safe way to augment posterior tracheal wall that is not uncommonly encountered after total laryngectomy (Figure 2). Combined with pectoralis major (PM) flaps, DP flap can reconstruct even some of the most extensive and complicated head and neck defects without using free microvascular flaps (Figures 3 and 4). When PM flap is used for anterior pharyngeal wall reconstruction, the nipple areolar complex is occasionally included to ensure adequate flap width to prevent neopharyngeal stenosis. Clear documentation in operation records is particularly important in such cases to avoid unnecessary anxiety in the future by mistaking the nipple as a lesion suspicious of recurrence.

In our experience, skin grafting of the donor site is necessary in most cases, although in theory skin laxity in older individuals may allow primary closure of smaller defects. In female patients, skin grafting the chest wall is cosmetically unpleasing and may lead to distortion of breast contour.

In our opinion, DP flap is indicated for patients with skin or mucosal defect in the head and neck region that required reconstruction but is not suitable for microsurgical procedure (e.g., lack of suitable recipient neck vessels due to previous treatments). DP flap is also indicated for management of complications, such as closure of salivary fistulas, release of neck contractures, or skin necrosis from radiation therapy. It is contraindicated when internal mammary artery has been compromised, for instance, from previous cardiac surgery.

## 5. Conclusion

Albeit the technical advancements in microvascular surgery and introduction of various new flaps, DP still possesses multiple unique advantages (technical simplicity, reliable axial blood supply, large size, thinness, and pliability) that few other reconstructive options can provide. Its versatility and reliability ascertain its role as an important reconstructive tool in well-selected patients.
